# Breast Density Should Play a Greater Role in MRI Screening Guidelines

**DOI:** 10.7759/cureus.37109

**Published:** 2023-04-04

**Authors:** Alan B Hollingsworth, Fang-Ying Li, Rebecca G Stough-Clinton

**Affiliations:** 1 Department of Screening Programs, Aurora Healthcare US Corp, Ijamsville, USA; 2 Department of Research, Aurora Healthcare US Corp, Ijamsville, USA; 3 Department of Clinical Imaging, Aurora Healthcare US Corp, Ijamsville, USA

**Keywords:** high-risk guidelines, breast density, breast cancer, screening epidemiology, breast mri

## Abstract

Guidelines for breast cancer screening with MRI were first proposed in 2007, only a few years after its clinical introduction. Those initial guidelines, which were generated by a committee sponsored by the American Cancer Society (ACS), have served as the template for similar recommendations by several organizations, with a singular goal regarding patient candidacy for MRI screening, a qualifying threshold based on risk stratification. Higher risk in those patients recommended for MRI screening translates to higher cancer detection rates, which in turn impacts cost-effectiveness.

But there is another variable that should be as important as risk stratification in selecting patients for MRI screening: the probability that screening mammography will fail to detect developing cancer. That failure rate is a function of breast density, included in the MRI screening guidelines as a traditional risk factor but neglected when one considers its role as the primary cause of false-negative mammograms. The two implications of dense mammograms are essentially independent: (1) refining risk stratification and (2) predicting the “miss rate” of mammography.

In the 2007 guidelines, indications for annual screening MRI, in addition to mammography, were based on patients having a calculated probability of “greater than 20-25% lifetime risk” for developing breast cancer. Other categorical risks, such as BRCA positivity, are listed in the ACS guidelines, but in effect, the threshold for adding MRI to the screening regimen has been a 20% lifetime risk for the development of breast cancer.

While risk stratification in the original MRI screening guidelines had a number of inconsistencies, the focus herein is the questionable placement of high-density patients into the category described as “no policy for or against MRI, more research needed,” a category where lifetime risks were grouped as 15-19%. Thus, mammographic density was relegated to its role as a traditional risk factor, while its potentially more significant impact, predicting the “miss rate” of mammography, had no role in patient selection for screening MRI.

The 2007 ACS guideline committee was limited by the lack of available data, and since there was no evidence for mortality reduction at the time, the decision was made to follow the patient selection criteria that had been used in the six international MRI screening trials, even though there was little consistency among those trials. Since then, the number of screening MRI trials has more than doubled, and new trials are being designed and implemented with a focus on both features of density: risk and cancer camouflage.

Enough evidence has accumulated during the 16 years subsequent to the original ACS high-risk screening guidelines to consider a complete revision that accounts for both numerical risk levels and density levels, much like what was used in the ACRIN 6666 trial. In establishing a new set of guidelines, our first question should be: What is the “miss rate” of mammography in this patient? If the chance of a false-negative mammogram is as high as we see with Level D density, then the decision to include MRI becomes straightforward. The traditional risk assessment would then be used to help determine the optimal interval between MRI screens while maintaining cost-effective cancer detection rates.

## Editorial

In 2007, when the American Cancer Society (ACS) became the first organization to address high-risk screening with breast MRI in addition to mammography [1], a conference call was held by the ACS to explain their rationale. In addition to adding annual MRI to annual mammography, the starting age for breast screening was lowered to 30 for high-risk women, a sharp departure from the “40 vs. 50” ongoing debate. Available evidence at that time included six international MRI screening trials wherein MRI showed vastly superior sensitivity to mammography, to the point that it was considered ill-advised to wait another decade or so before mortality reductions could be confirmed.

On that conference call, there was no criticism regarding the introduction of breast MRI. Rather, the questions were focused on proper patient selection and what some participants believed were inconsistencies and nuanced problems that would arise based on known principles of risk assessment.

We previously addressed one of those inconsistencies [2] wherein the use of remaining lifetime risk discriminates against older women who are entering peak short-term incidence for developing breast cancer, a problem easily averted if short-term risk calculations are added to current lifetime approaches. Remaining lifetime risks decline over time, while short-term incidence increases, and this paradox has generated a great deal of confusion in risk assessment for both MRI screening criteria and pharmacologic risk reduction.

After multiple concerns were raised during the conference call with the ACS guideline committee, the speaker finally stated, “Remember, these are just guidelines.” Of course, guidelines have a way of morphing into rules, and it was not long before peer reviewers were denying insurance coverage for screening breast MRI based on their risk calculations.

A countermeasure came from physicians and healthcare providers who adopted the IBIS (Tyrer-Cuzick) model given its propensity for generating higher risk numbers than other models. Although mentioned in the ACS report, the Tyrer-Cuzick model was not their first choice, based on the approaches that had been used in international MRI trials. At the time, breast cancer risk assessment was relatively new to clinicians, its earlier use having been limited to patient selection for pharmacologic risk reduction, which was not a pervasive practice. In fact, it can be argued that the widespread interest in breast cancer risk assessment was largely generated by the 2007 MRI screening guidelines.

In those guidelines, the ACS held that the risk models to be used should be “BRCAPRO or those models focused primarily on family history,” somewhat open-ended given that all models at the time focused on family history. Furthermore, tables presented in the guideline article included widely disparate calculations for risk depending on the chosen model, adding to the awareness that one could utilize several approaches and then pick the one with the highest calculated risk for reimbursement purposes. Given that there are legitimate strengths and weaknesses for each model, this practice of ramping the risk as high as possible has not always served the best interest of our patients. Exaggerated risk levels can prompt some patients to abandon high-risk surveillance with MRI and opt for preventive surgery instead.

In their defense, the ACS guideline committee was simply trying to follow what had been utilized for patient accrual in the six international trials of breast MRI screening available at that time, a difficult task since there was a wide range of approaches, from a 15% lifetime risk threshold (the Netherlands) to one trial limited exclusively to BRCA-positive patients (Canada).

The most basic, as well as the most common, criterion for breast MRI screening was a “lifetime risk of 20-25% or greater,” which, in practice, is the same as “greater than 20%.” Less commonly encountered indications for MRI at the time were BRCA-positivity, an untested first-degree relative of a BRCA-positive patient, several rare genetic disorders, and radiation therapy to the chest between age 10 and 30, any one of which would place patients in excess of 20% lifetime risk. Thus, the pre-eminent feature in qualifying for screening MRI was the 20% threshold. For those who calculated below 15% lifetime risk, MRI was specifically not recommended (in sharp contrast to 15% being the threshold for MRI screening in the Netherlands trial).

Most problems centered in the middle group, with 15% to 19% lifetime risk (“insufficient evidence for or against MRI”), a category where atypical hyperplasia and lobular carcinoma in situ (LCIS) were placed. Thus, a young patient with atypical hyperplasia might calculate a 40% remaining lifetime risk, generating conflicting recommendations since the “20%” threshold was contingent on this risk being due to family history. Thus, a young patient with a modest family history and a lifetime risk of 21% would qualify for MRI but not the same patient at 40% if the risk was due to atypical hyperplasia or LCIS. This paradox was due to the fact that none of the international screening trials had used tissue risks (atypical hyperplasia, LCIS) for accrual. Therefore, the ACS committee was reticent to endorse those risk factors.

As it turned out, since atypical hyperplasia and LCIS are included in the Tyrer-Cuzick model, most practitioners went ahead with MRI screening if the final lifetime risk calculation exceeded 20% (even though the Tyrer-Cuzick model has been criticized for overestimating the risk imparted by atypical hyperplasia and LCIS). Later on, other medical societies that adopted the general principles of the ACS guidelines recognized this paradox and issued guidelines more consistent with general practice, accepting risk levels regardless of the specific factors behind the elevated risk.

However, risk stratification as an indication for MRI screening does not work so well for breast density. This is because there is a unique variable at play in addition to the risk imparted by the density; that is, density dictates the probability that a developing cancer will be camouflaged, generating a higher “miss rate,” that is, false-negative mammograms. This feature is distinct from density in its role as a risk factor for future breast cancer.

The ACS chose to focus on the breast cancer risk imparted by Level C and D density, placing it in the “insufficient evidence” category where the lifetime risks were 15-19%. However, if one is considering a second screening tool added to the first line tool, wouldn’t the most critical question be: “What is the probability that the first modality will fail?” This question is not answered through risk factors. In fact, one can make the case that this additional feature of density, a higher “miss rate,” is more impactful than the risk alterations imparted by density.

For instance, if the patient has a fatty replacement on mammography as the background, is there really any need for supplementary imaging? In contrast, if a patient has extreme density on mammography, how important is the general risk assessment if the false-negative rate with mammography alone is so high in this group of patients?

When we started our screening MRI program in the pre-guideline era (2003), it seemed that the question of breast density was as important as calculated risk levels. Thus, we used a scoring system that gave equal importance to the calculated risk and breast density levels.

As it would turn out, the designers of the ACRIN 6666 trial [3] had the same idea, with entry into their multimodality imaging study (US and MRI, in addition to mammography) requiring both features, risk and density, and these two values were equally weighted. The protocol was so refined in this regard that the two variables interacted; that is, a higher density level prompted a lowering of the risk threshold for entry and vice versa. This showed a clear understanding of the fact that density has two distinct implications: (1) the risk of developing cancer and (2) the risk that developing cancers will be missed on mammography. Only the former is considered in ACS guidelines. Somehow, breast density as a risk factor stole the show, leaving the serious implication of failure to detect cancer untouched.

With the inclusion of breast density into version 8.0 of the Tyrer-Cuzick model, it might seem that the problems noted above have been addressed. However, that is not exactly the case as the model has simply addressed the imparted risk of dense breasts, not the more worrisome “missed cancer,” i.e., false-negative mammograms. A common belief holds that version 8.0 improves access to MRI as more women will qualify if they have dense breasts, but this impact is limited. Women with Level A and B density have a reduced breast cancer risk with version 8.0, possibly taking them below the 20% threshold that would have otherwise been reached. Level C density is very close to being the neutral referent (RR=1.0), with minimal change in absolute risk as calculated by version 8.0. Only Level D patients (10% of mammograms) experience a substantial increase in calculated risk with the latest version of the Tyrer-Cuzick model. The vast majority of patients either remain at the same level of risk or have a lowered risk than was calculated by version 7.0. For Level D patients, it can be stated that access to MRI is “improved,” thus indirectly covering that second feature of density, the miss rate.

Appreciation of the camouflaging impact of density can be seen in the ongoing DENSE Trial Study Group in the Netherlands [4] where the only entry requirement for participation in this MRI screening study is Level D mammograms (extreme density). Other risk factors are not required. In spite of low compliance (59%) in those randomized to an invitation to undergo MRI screening, this prospective, randomized trial has reported a statistically significant lowering of interval cancers, the primary endpoint, while mortality data will be forthcoming.

In our point system that started in 2003, equal weight was given to density levels and risk levels (four density levels and three risk levels), and we tracked outcomes drawn from our pre-existing high-risk clinic. It was clear early on that nearly all of our MRI-discovered cancers were coming from the high-density group, with or without risk factors. In addition, when the 2007 guidelines were announced, we saw that the majority of our cancer discoveries would not have qualified for MRI had we followed those guidelines. When we last reported on our experience with this cohort [5], using the most liberal interpretation of the ACS guidelines for MRI screening, we would have identified only 16 of 33 (48.5%) patients with mammographically occult cancer.

Twenty years ago, our point system also introduced the options of biennial and triennial screening with MRI. Today, recommendations in the United States remain all-or-nothing. Therefore, the patient at 21% lifetime risk is recommended for an annual MRI, the same interval as the BRCA-positive patient at 80% lifetime risk. Instead, drawing from the efficacy of mammography at intervals of two years and longer in the historical mammography screening trials, it seemed reasonable to entertain longer intervals between MRIs for those at the lower end of risk/density continuums.

As time passed, however, and the ACS guidelines became closely tied to reimbursement, our MRI screening program had fewer qualifiers for MRI, and thus fewer cancers were discovered. Although our cancer detection rate remained the same (1.8%), the total number of MRI-detected cancers was cut by more than half. The patients who had been previously diagnosed through MRI based on density were no longer qualified, an unfortunate turn of events, somewhat mollified by our shift to using whole breast ultrasound for these patients.

In addition, based on the work of Dr. Christiane Kuhl who has been using MRI to screen the general population at baseline risk, as well as the preliminary findings from the DENSE trial in the Netherlands, the European Society of Breast Imaging has recently recommended MRI screening in women with extremely dense tissue (Level D), independent of other risk factors, to be performed every two to four years. This marks the first time the “miss rate” in high-density patients has driven MRI screening guidelines.

Although we considered our 2003 biennial and triennial intervals to be somewhat risky at the time, the available evidence now points to very few interval cancers emerging if the new European Society of Breast Imaging guidelines of “MRI every two to four years” is followed for the Level D patients.

Currently, breast MRI screening guidelines in the United States emanate from several organizations, differing only by minor variations. However, they are all derivatives of those initial ACS guidelines in 2007 that included paradox and inconsistencies while neglecting the full impact of breast density.

Subsequent to the 2007 ACS guidelines, the number of published breast MRI screening trials more than doubled. In addition, international trials are underway that recognize and include breast density because of its association with the “miss rate” in addition to the imparted risk for developing breast cancer. The stage is set for new MRI screening guidelines, this time based on individualized density levels that predict the potential benefit of MRI, along with risk stratification that optimizes cancer detection rates.
